# Characterization of undifferentiated human bone marrow and blood derived mesenchymal stem cells and their potential for chondrogenic differentiation

**Published:** 2011-01-10

**Authors:** Pan-Pan Chong, L Selvaratnam, T Kamarul

**Affiliations:** 1 Departments of Orthopaedic Surgery & University of Malaya, 50603 Kuala Lumpur, Malaysia; 2 School of Medicine & Health Sciences, Monash University, Sunway Campus, 46150 Subang Jaya, Selangor, Malaysia

## 

In recent years, the potential of cartilage tissue engineering techniques employing mesenchymal stem cells (MSCs) to repair damaged human cartilage and defects has generated much interest. Traditionally, sources of MSCs included patient’s own bone marrow. However, little have been reported on adult peripheral blood (PB) as a source of MSCs, which has been a subject of much debate amongst scientists owing to its extremely low density in PB and the difficulties associated with extracting MSCs from PB. The objectives of this study were to isolate MSCs derived from bone marrow and peripheral blood as a source and to assess their potential to undergo *in vitro* chondrogenesis using a biocompatible three-dimensional scaffold. In defining MSCs, the cells isolated from its source must meet these 3 criteria: (i) adherence to plastic when maintained in culture; (ii) positive expression of several antigens such as CD29, CD105, CD166; (iii) ability to differentiate into osteoblasts, adipocytes & chondrocytes under *in vitro* inductive conditions. PB samples (2 ml) were collected and mononuclear cells extracted and separated using Ficoll–Paque PLUS via centrifugation. Subsequently, suspended cells were removed after 5 days of culture, and adherent cells left to grow. Cells were detached upon reaching 80-90% confluence and sub-cultured up to 4 passages prior to further experiments. MSC antigens were recognized by monoclonal antibodies CD29, CD105 and CD166. To distinguish MSCs from hematopoietic stem cells, CD34 surface markers were used as negative controls. The characterized human blood-derived progenitor cells were cultured in three-dimensional alginate scaffolds using chondrogenic induction medium to promote chondrogenesis. Chondrogenesis was quantitated by sulphated glycosaminoglycan (S-GAG) production measured by 1,9-dimethylmethylene blue (DMMB) assay. Furthermore, chondrogenic-MSCs were examined and histologically compared using Safranin O staining to that of human chondrocytes as a means to determine chondrogenic transformation. Gene expression analysis was carried out by reverse transcriptase-polymerase chain reaction (RT-PCR) of differentiated human blood-derived progenitor cells using chondrocyte (cartilage cell)-specific phenotypic markers. The results showed that the cell derived in our processing technique share similar characteristics with adult MSCs and chondrocytes. Induction of chondrogenesis has been demonstrated in human blood-derived progenitor cells, which could provide a ready source of chondrocytes for engineering biological therapies. In the practical sense, PB isolation would prove to be less invasive, less expensive and less traumatic for patients to undergo therapy as compared to the 2-stage procedure of current available tissue engineering technique.

